# Poxviral Ankyrin Proteins

**DOI:** 10.3390/v7020709

**Published:** 2015-02-15

**Authors:** Michael H. Herbert, Christopher J. Squire, Andrew A Mercer

**Affiliations:** 1 School of Biological Sciences, University of Auckland, Auckland 1010, New Zealand; E-Mails: mher065@aucklanduni.ac.nz, (M.H.H.); c.squire@auckland.ac.nz (C.J.S.); 2 Virus Research Unit, Department of Microbiology and Immunology, University of Otago, Dunedin 9016, New Zealand

**Keywords:** poxvirus, ankyrin, F-box, ubiquitin, NF-κB, phylogenetics, evolution

## Abstract

Multiple repeats of the ankyrin motif (ANK) are ubiquitous throughout the kingdoms of life but are absent from most viruses. The main exception to this is the poxvirus family, and specifically the chordopoxviruses, with ANK repeat proteins present in all but three species from separate genera. The poxviral ANK repeat proteins belong to distinct orthologue groups spread over different species, and align well with the phylogeny of their genera. This distribution throughout the chordopoxviruses indicates these proteins were present in an ancestral vertebrate poxvirus, and have since undergone numerous duplication events. Most poxviral ANK repeat proteins contain an unusual topology of multiple ANK motifs starting at the *N*-terminus with a *C*-terminal poxviral homologue of the cellular F-box enabling interaction with the cellular SCF ubiquitin ligase complex. The subtle variations between ANK repeat proteins of individual poxviruses suggest an array of different substrates may be bound by these protein-protein interaction domains and, via the F-box, potentially directed to cellular ubiquitination pathways and possible degradation. Known interaction partners of several of these proteins indicate that the NF-κB coordinated anti-viral response is a key target, whilst some poxviral ANK repeat domains also have an F-box independent affect on viral host-range.

## 1. Poxviruses

Poxviruses are nucleocytoplasmic large DNA viruses (NCLDV) [1,2] that replicate exclusively in the cytoplasm and maintain genomes of 130 to >350 kilobases. They infect a wide range of vertebrate and invertebrate hosts and are divided into two sub-families: the *Chordopoxvirinae*, which infects vertebrate hosts (reptiles, mammals and birds), and the *Entomopoxvirinae*, which infect invertebrates (insects) [3]. The chordopoxviruses consist of 10 classified genera and contains the most well-known and well-studied species, including the infamous causative agent of smallpox, variola virus, as well as the non-virulent vaccinia virus that enabled the global eradication of smallpox by vaccination [4,5]. Poxviral infections can exert significant burdens in livestock and wild animal populations and can produce dramatic effects in new host species. This is most vividly shown by the myxoma poxvirus, which produces the fatal disease of myxomatosis in European rabbits but not in its natural host species, American rabbits [6]. Several poxviruses are zoonotic but have a limited impact on human health with low virulence, the main exception being the historical influence of smallpox with its high morbidity and mortality. Contemporary infections of humans by poxviruses are generally limited in immunocompetent individuals, although the monkeypox virus does have a low mortality rate in zoonotic infections [7,8,9].

The genomic content and organisation is highly similar across poxviruses, and especially within the chordopoxviruses. Genes required for poxviral DNA replication and transcription, along with those involved in virion morphogenesis and structure are typically located within a highly conserved central region of the poxviral genome. Comparative analysis of published poxviral genomes demonstrates that 90 genes are conserved in all poxviral species, of which approximately 40 are conserved only in the chordopoxviruses [10,11,12]. There is also a well-conserved synteny between these genes in most chordopoxviruses, but this is reduced in the avipoxviruses, and does not extend to the entomopoxviruses [13]. Although the protein similarity within genera appears sufficient for effective vaccination by cross-protection between species [14,15], this does not seem to apply between genera [16]. The terminal regions of the linear poxviral genome introduce a significant variation between poxviral species and are replete with a wide range of genus- or species-specific genes. Proteins expressed from these terminal regions frequently determine host-range and also significantly contribute to infection and virulence by successfully interfering with the cellular and immune response to viral presence [17,18,19,20].

Located among the terminal regions of the majority of chordopoxviral genomes are genes coding for multiple proteins rich in ankyrin repeats (ANK), 33 residue-repeating motifs commonly associated with protein-protein binding. ANK repeat proteins are highly abundant in the *Avipoxvirus* genus, with 26 ANK repeat proteins detected in a pigeonpox virus (FeP2 strain), 31 present in fowlpox virus (Iowa strain), and 51 found in canarypox virus (ATCC VR111 strain) [21,22,23]. ANK repeat proteins become less prevalent within the *Orthopoxvirus* genus ranging from ten within variola virus (Bangladesh 1975 strain) to 15 within cowpox virus (Brighton Red strain) [17,24,25,26,27,28]. ANK repeat proteins are less numerous again within the *Capripoxvirus*, *Leporipoxvirus*, *Suipoxvirus*, *Yatapoxvirus* and C*ervidpoxvirus* genera [29,30,31,32,33] referred to as the Leporipoxvirus super-group (LSG) [34], or alternatively as clade II genera (based on their clustering separately from the clade I orthopoxviruses) [35]; a similar number are also present within genomes of the *Parapoxvirus* genus [36,37,38], and the unclassified cotia virus species [39]. ANK repeat proteins are absent from the known species of *Molluscipoxvirus*, *Crocodylidpoxvirus* and the red squirrel poxvirus [34,40,41,42,43] and from all the known *Entomopoxviru*s genera [44,45,46].

## 2. The Ankyrin Repeat Motif

The ANK repeat motif is known to be one of the most abundant in nature [47]. The Pfam database contains 134027 sequences representing 3000 species within the ANK repeat super-family (Table 1). The majority of these predicted ANK repeat proteins are found in eukaryotic species, whilst bacterial representatives are not as common, and even fewer are found in archaeal species; viral ANK repeat proteins are mainly, but not exclusively, limited to the poxviruses [48]. Furthermore, the range of different sequences of ANK repeat proteins is greater throughout the eukaryotes, with less variation seen within the other kingdoms.

**Table 1:** Pfam ankyrin groups. The ankyrin repeat superfamily (Pfam clan CLO465) contains several groups of ANK repeat proteins. Species and sequence distribution, along with numbered of determined crystal structures, amongst the groups for all species, and for poxvirus species only.

Pfam Ankyrin Group	Pfam Identifier	All Species			Poxvirus Only		
		Species	Sequences	Structures	Species	Sequences	Structures
ANK1	PF00023	897	8812	214	42	247	0
ANK2	PF12796	2889	110723	200	45	478	1
ANK3	PF13606	202	446	0	8	12	0
ANK4	PF13637	729	7425	31	18	56	0
ANK5	PF13857	768	5157	6	12	43	0
ANK6	PF11900	51	221	0	0	0	0
ANK7	PF11929	1	1243	0	0	0	0

The initial identification of the ANK repeat unit and the first indications of its widespread occurrence came when it was described as a two-repeat motif found within the yeast cell cycle control proteins Sw16 and Cdc10 [49], which also showed consensus to a motif within the Notch protein of *Drosophila melanogaster* that has five such repeats. This motif was later discovered in, and named after, the human erythrocytic ankyrinR protein [50] that contains 24 repeats of the motif. AnkyrinR links membrane-associated proteins, including ion channels and transporters, and cell adhesion molecules, via its ANK repeat motifs to the cell’s spectrin cytoskeleton scaffold using a spectrin binding domain [51,52,53]. The canonical ankyrinR protein family is not found outside of metazoan organisms, and is believed to have evolved prior to the separation of arthropod and vertebrate lineages, forming an essential component of the support structure for the eukaryotic cell membrane [54,55]. Consensus searches using the ankyrinR protein identified the first ANK repeat units within vaccinia and cowpox viral proteomes [50], with the initial characterisation of ANK repeat protein distribution within poxviral genomes following shortly thereafter with the publication of the vaccinia and variola major virus genomes [26,27,28].

**Figure 1:**
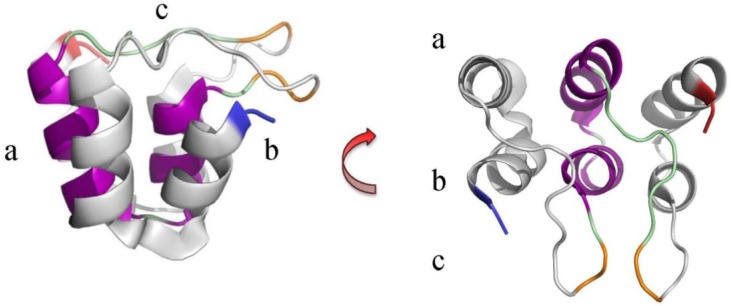
The ANK repeat unit from the ankyrinR protein. Example of an ANK motif highlighting the 5^th^ repeat unit in the ankyrinR structure (PDB ID: 1N11) [52]. β-turns (orange) within the loops (green) link the helix pairs and project outwards in a conserved manner at an angle of approximately 90° from the α-helices (purple) [47,52,56]. This arrangement has been likened to a “cupped hand” where the convex surface forms the “back” (**a**)’ the concave surface the “palm” (**b**)’ and the loops form the “fingers” (**c**). Blue and red indicate the *N*- and *C*-termini respectively.

The ANK motif structure comprises two short α-helices connected by β-turns and short loops, and often forms a series of repeats where the β-turn/loop regions align and project away from the α-helices at 90° (Figure 1). This configuration, together with one α-helix (the inner helix) being slightly shorter than the other (the outer helix), produces a distinctive curved structure with a defined outer convex surface and an inner concave surface that is cupped by the β-turn/loops and inner α-helices. The ANK repeats may also be slightly rotated with respect to each other, so in the ankyrinR structure (PDB: 1N11) there is a 2°–3° turn per repeat, contributing to the super-helical form, a complete turn of which would need 32 repeats [47,52].

**Figure 2:**
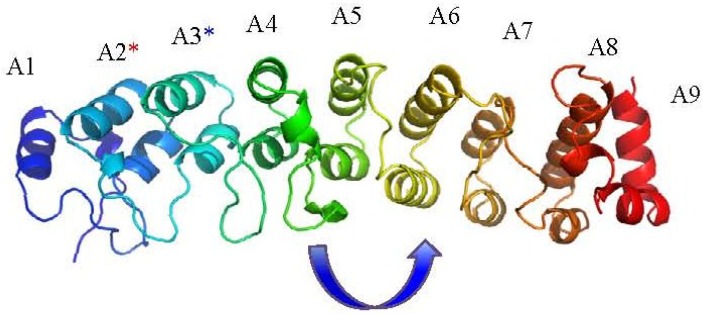
The vaccinia virus K1L ankyrin protein structure (PDB ID: 3KEA) [58]. ANK repeats are numbered A1-A9; the terminal ANK repeat motifs 1 and 9 are in-complete motifs in the structure. The blue asterisk refers to residues F82 and S83 on A3 [59] required for *in vitro* viral replication; the red asterisk refers to residues C47 and N51 on the A2 repeat that are spatially contiguous with F82 and S83 and are also required for host-range function. The arrow indicates a surprising 90° shift in the orientation of β-turn loop arrangement from repeats A4 to A5.

Functional analysis has confirmed that the ANK repeat has a structural or regulatory role, rather than an enzymatic one, though it may often be found associated as part of multi-domain proteins, or as part of a multi-protein complex [47,52,57]. The ability to bind proteins is not restricted to a conserved sequence within the consensus ankyrin repeat, but rather it is due to the orientation of the repeat units creating accessible binding sites. These are derived from the arrangement of residues separated in sequence position being brought together to create larger binding patches on the concave or convex surface of the ankyrin protein molecule [57,58]. Examples of structural, and biophysical, characterisation of poxviral ANK repeat proteins are unfortunately scarce, with only the crystal structure of vaccinia virus K1L protein (PDB ID: 3KEA) [58] confirming the presence of the sequence-predicted poxviral ANK motif repeats, and demonstrating the characteristic extended helical form of an ANK repeat protein due to the stacking of the multiple motifs (Figure 2). This structure revealed several unexpected features for this protein with the critical residues known to affect host-range function exposed on the convex surface “back” of the stacked repeats, rather than the concave “palm” usually associated with ANK mediated interactions [47,58]; the authors suggest this may indicate a more transient interaction with host-proteins. There is also an interesting shift in the position of the β-turn loops of approximately 90° after the A4 motif, and, additionally, the entire sequence is composed of ANK repeats, including only partial versions of the ANK motifs at the *N*- and *C*-termini [58].

## 3. Ankyrin Proteins in Eukaryotes and Prokaryotes

In eukaryotes the ANK motif is thus found associated with many aspects of protein-protein interaction, demonstrating an essential role in eukaryotic cells connecting many regulatory and structural functions [47,56]. Examples of these include scaffolding interactions with the multi-domain ankyrin group, and also the SHANK proteins that contribute to post-synaptic density in neurons, [60,61]. Ankyrin motifs are also associated with proteins involved in intra-cellular signalling, such as the IκB protein, the ANK repeats of which sequester nuclear factor-κB (NF-κB) to regulate gene transcription [62,63], the intra-cellular domain of the Notch receptor, which is essential in signalling of transcriptional regulation of cell-cycle processes [64] and the gankyrin oncoprotein, which is composed entirely from seven ANK repeat motifs and that has multiple binding partners related to cell-cycle control [65] Additional multi-domain eukaryotic proteins include the tankyrase 1 and 2 proteins, that contain 22 ANK repeats along with a poly(ADP-ribose) polymerase domain and are involved in telomere protection and replication [66] the KANK proteins involved in regulating actin polymerisation [67] the MARP proteins associated with signalling and transcription regulation in muscles [68] and the ANK repeat containing transient receptor protein (TRP) channels involved in sensory signal transduction [69].

With such a multitude of roles ANK repeat proteins have been estimated to comprise ~0.8% of eukaryotic proteomes. An average of 125 ANK repeat proteins are found in any given eukaryotic proteome, but are more prevalent than in bacteria, where they represent approximately 0.1% of the proteome; ANK repeats are even rarer in archaea, at ~0.07% prevalence [48]. However, ANK repeat proteins are enriched in intracellular bacteria that have an obligate or facultative symbiosis with the host, and can constitute to up to 4% of the proteome. Bacterial ANK repeat proteins are found in several intracellular pathogens including *Anaplasma phagocytophilum* (anaplasmosis in sheep, cattle and humans), *Ehrlichia chaffeensis* (human monocytotropic ehrlichiosis), *Orientia tsutsugamushi* (scrub typhus), *Legionella pneumophila* (Legionnaire’s disease), *Coxiella burnetii* (Q fever), as well as the obligate endosymbiont *Wolbachia pipientis* [70]. ANK repeat proteins are surprisingly more abundant in poxviruses and can range in prevalence from 2% in parapoxviruses and the LSG group proteomes, to up to 8% in some orthopoxvirus species, and even higher in the avipoxvirus species representing 12% of the fowlpox virus and 15% of canarypox virus proteomes.

## 4. Poxviral Ankyrins and F-Boxes

**Figure 3:**
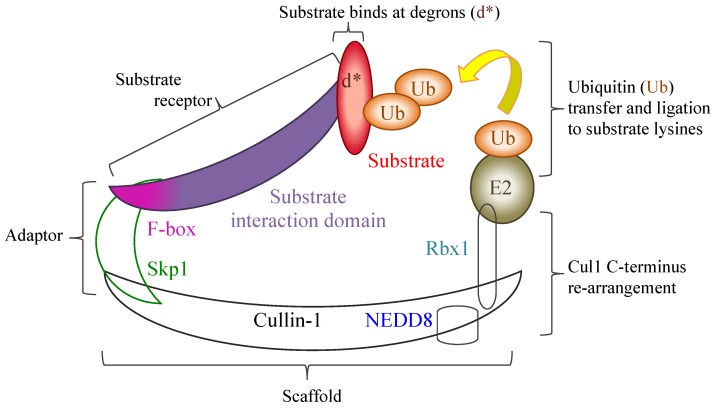
Representation of a cellular SCF1 ubiquitin ligase complex following neddylation, where NEDD8 ligation to the Cul1 scaffold facilitates a re-configuration of the Cul1 *C*-terminus. This enables the flexible re-orientation of the Rbx1 RING domain, together with its bound E2 and an associated ubiquitin allowing the subsequent transfer and ligation of ubiquitin at specific lysine residues in the substrate or previously bound ubiquitins. Poxviral ANK/F-box proteins are known to bind to Skp1, via their PRANC/F-box region, and therefore could replace cellular substrate receptors and interact with possible substrates using their ANK repeats as the substrate interaction domain.

Poxviral ANK repeat proteins are usually large ranging in size between 400 and 800 residues in length, and retain a characteristic domain arrangement, whereby the ANK repeat motif is present in multiple contiguous repeats numbering between four and ten, starting from the *N*-terminus. The ankyrin repeats are usually followed by an apparently non-ankyrin linker sequence which ends at the *C*-terminus in a motif that has sequence homology to the cellular F-box [71]; this arrangement is annotated in the Pfam database as the PRANC domain (Pox protein repeats of ankyrin—*C*-terminal, Pfam family PF09372). The cellular F-box motif is associated with ubiquitin ligase E3 complexes involved in ubiquitin tagging of specific protein substrates for degradation via the 26S proteasome or other fates [72,73]. The largest class of ubiquitin ligases consists of multi-subunit complexes built on the large helical cullin protein scaffolds. F-box proteins are a component part of the SCF1 complexes (Skp1, Cullin1, Rbx1, F-box) [74], which ligates ubiquitin to a wide variety of substrates selected by numerous different F-box proteins. In cellular F-box proteins the F-box motif is typically located near the *N*-terminus, and connect by a short linker sequence to the substrate binding domain, which is frequently a multiple-repeat sequence such as a leucine rich repeat or a WD-40 motif [75]. The F-box motif connects to the SCF1 complex via the Skp1 adaptor protein, which binds to the Cul1 protein *N*-terminus. Cul1 acts as scaffold and also binds the RING (really interesting new gene) protein, Rbx1, at its *C*-terminus (Figure 3). The targeted substrate protein binds via its specific degradation sequences (degrons) to the F-box substrate receptor but remains separated by at least 50 Å from the Rbx1-bound ubiquitin-conjugating enzyme, E2, bearing the ubiquitin tag for ligation. Activation of the complex requires a step known as neddylation, where an ubiquitin-like protein NEDD8, is ligated to the cullin at a lysine in the *C*-terminal half leading to structural re-configuration of the cullin *C*-terminus and its bound Rbx1. This re-arrangement induces greater flexibility in the orientation of the RING domain of Rbx1 and its associated E2-ubiquitin, moving these sufficiently close enough towards the substrate receptor bound target to allow its initial and poly-ubiquitination [76,77].

The poxviral F-box is truncated in comparison to the cellular version, containing only two of the three short α-helices that constitute the cellular F-box [78]. Biochemical analysis has demonstrated that this does not impede interaction with Skp1 or the larger SCF1 complex, and many poxviral ANK repeat proteins can associate with these cellular proteins via their truncated F-box motifs [78,79,80,81,82,83,84,85]. Indeed, one cowpox virus ANK/F-box protein, CP77, can interact with Skp1 even though its F-box is effectively reduced to just the first α-helix [79]. The interaction between poxviral F-boxes and their protein partners, represented by examples from orf virus ANK/F-box proteins, has been modelled as very similar to that known for cellular proteins [86]. Structural assessment of F-box interactions show that the majority of the atomic interactions are by van der Waals bonds and hydrogen bonds that derive from within the first α-helix and a short loop that precedes it [87,88], therefore it is not surprising that the truncated poxviral versions can maintain their partner interaction and thus probably compete with the large variety of host F-box proteins. However, the strength of the affinity between cellular F-box proteins and their Skp1 adaptor are not yet fully understood. An absence of the F-box region is prominent in one group of orthopoxvirus ANK repeat proteins that have a host-range function, represented by the vaccinia virus K1L protein [89,90]. Unlike other poxviral ANK repeat proteins that lack the *C*-terminal F-box this is not believed to be due to deletion or truncation events of intact comparable proteins ANK/F-box sequences [34,40]. ANK motifs have not been observed in cellular F-box proteins, although they do occur in a similar E3 ubiquitin cullin-5 ligase complex that uses a SOCS (suppressor of cytokine signalling) box rather than an F-box [91].

## 5. Poxviral ANK/F-Box Protein Phylogenetics

Two separate phylogenetic analyses of the ANK/F-box protein distribution within the *Chordopoxvirinae* have been completed showing that the majority of the ANK repeat proteins can be distinguished from each other within each poxvirus species [34,40]. The ANK repeat proteins form orthologue groups spanning multiple species within a genus, or in the case of the LSG group across both species and genera. This distribution mirrors the phylogeny seen for poxviruses in general, where *Avipoxvirus*, *Parapoxvirus*, and *Orthopoxvirus* genera can be clearly distinguished from each other, with the LSG group clustering together to form their distinct clade [13,35,40,92,93]. The ANK repeat protein orthologue groups thus form a series of clades reflecting likely duplication events from the ancestral poxviral ANK repeat proteins. The *Orthopoxvirus* and LSG group contain sufficient sequence similarities to definitively show these were descended from a shared lineage. The avipoxvirus and parapoxvirus ANK repeat proteins are postulated to have arisen from earlier separation events in the ancestral chordopoxvirus lineage with subsequent genus-specific duplications generating clades of their own [40]. Table 2 details the ANK repeat gene locations in either or both genomic termini, and whether they are intact or disrupted by truncations or insertions; the ANK repeat gene names are based on their deposited genomes and their subsequent phylogenetic analysis [17,25,26,27,30,31,32,33,34,38,39,40].

In the classification of Bratke *et al.* (2013) [40] the expansion of ANK repeat protein numbers was explored more fully with the *Orthopoxvirus* and LSG genera. This identified 13 duplication events that have resulted in 14 different clades being recognised for intact ANK repeat proteins that contain the *C*-terminal F-box, with these proteins separating into two major groups, clades 1 to 5 and 6 to 10 in the orthopoxviruses. Table 2 shows the distribution amongst four key orthopoxvirus species: cowpox virus, Brighton Red strain (CPXV-BR) [17,34,40]; variola virus, Bangladesh 1975 major strain (VARV_B75) [27,34,40]; ectromelia virus, Moscow strain (ECTV_Mos) [25,40]- the Naval strain was assessed in Sonnberg *et al.*, 2011 [34]; vaccinia virus, Western Reserve strain (VACV_WR) [40]—the Copenhagen strain was assessed in Sonnberg *et al.*, 2011 [26,34]. The ancestral orthopoxvirus ankyrin protein that resulted in the groups 6 to 10 was also present in the LSG genera and underwent duplication there to form the distinct groups 11 to 14, along with a maintenance of several LSG ANK repeat proteins (exemplified by the deerpox virus DPV019 protein) that bear a closer relationship with the orthopoxvirus group 6. In the Sonnberg *et al.* (2011) orthologue classification [34], which contains both full-length and truncated proteins, the identified phylogenetic groups were numbered within each genus (counting the LSG genera as a single clade) with respect to the gene position in the linear genome from left to right (Table 2). This also identified ten groups of intact ANK repeat proteins in the *Orthopoxvirus* genus plus five groups of truncated proteins. An additional two groups were also identified in the LSG genera, with truncated proteins being mainly limited to LSG group II, represented by DPV014.

The distribution of orthologues within the LSG is shown for four species in Table 2: myxoma virus, Lausanne strain (MYXV_Lau) [31,34,40]; sheeppox virus, TU-V02127 strain (SPPV_Tu) [33,34,40]; tanapox virus, Davis strain (TPV_Dav) [32,34]- the Kenya and RoC strains were used in Bratke *et al.* 2013 [40]; and deerpox virus, W1170-84 strain (DPV_84) [30,34,40]. Seven orthologue groups were identified within the parapoxvirus species of orf virus strain, NZ2 strain (ORFV_N2), and bovine papular stomatitis parapoxvirus, BV-AR02 strain (BPSV_AR02), without any truncated versions present [34,36,37]. BPSV contains two additional copies of ANK repeat genes, BPSV002 and BPSV003, compared to orf virus and also to pseudocowpox virus, which otherwise all share a similar synteny in the ANK repeat gene position [34,37,38].

The *Avipoxvirus* genus contains significantly more ANK repeat proteins, as well as greater rearrangement in location between species, and less similarity between proteins. A definitive numbering was not attempted with only one likely orthologue group shared between fowlpox virus and canarypox virus (containing FPV244 and CNPV009 at 81% identity) and a further possible 16 groups of lower amino acid identity between 40%–60% identity [34], a significant number of truncated forms were also present with 8 of 31 ANK repeat proteins in fowlpox virus truncated and 15 of 51 canarypox virus ANK repeat proteins. Similar levels of ANK repeat protein truncation and fragmentation were also reported for penguinpox virus and pigeonpox virus [22].

**Table 2:** Orthologue groups of poxviral ANK repeat proteins of key species and strains. Location and identity of ANK repeat genes in genomic termini (L, left, R right) are shown, with truncated or disrupted genes in grey (Tr). Protein orthologue groups are based on Sonnberg, *et al.* (2011) [34] and Bratke, *et al.* (2013) [40]. Well-studied proteins or homologues are indicated: ^a^ CWPX-CP77, ^b^ VACV-K1L, ^c^ VACV-68k, ^d^ VARV-G1R.

Poxvirus Genus, Species_Strain, and Location for ANK Repeat Genes						ANK Repeat Protein Orthologue Groups	
Genome Terminus		Orthopoxvirus				Sonnberg	Bratke
		CPXV_BR	VARV_B75	ECTV_Mos	VACV_WR	2011 [34]	2013 [40]
L		006/225	-	002/171	005–008	I	5
L		008/223	D1L	-	-	II	10
L		011	-	005	-	III	2
L		016	-	010	-	IV	Tr
L		017	-	-	-	V	8
L		019	-	-	-	VI	Tr
L		025^a^	D8L	-	014–017	VII	7
L		027	-	-	019	VIII	9
L		039	O1L	021	030	IX	Tr
L		041 ^b^	C1L	022	032	X	Tr
	R	198	B5R	154	186	XI	6
	R	200	-	-	188	XII	Tr
	R	211 ^c^	B16R	-	199	XIII	3
	R	213	B18R	165	202	XIV	1
	R	220	-	-	-	XV	4
	R	008/223	-	-	-	II	10
	R	006/225 ^d^	G1R	002/171	211-214	I	5
		Leporipoxvirus super-group (LSG)					
		MYXV_Lau	TPV_Dav	SPPV_Tu	DPV_84		
L		m005L	-	-	-	I	14
L		-	8L	010	014	II	Tr
L		-	11L	-	019	III	OPXV 6
	R	m148R	-	138	161	IV	12
	R	m149R	146R	140	164	V	13
	R	-	147R	141	165	VI	11
	R	m150R	148R	145	166	VII	14
	R	m005R	-	-	-	I	14
		Parapoxvirus					
		ORFV_NZ2		BPSV_AR02			
L		-		002		I	n/a
L		-		003		II	n/a
L		008		008		III	n/a
	R	123		123		IV	n/a
	R	126		126		V	n/a
	R	128		128		VI	n/a
	R	129		129		VII	n/a

Cowpox virus (Brighton Red strain) is the only orthopoxvirus species that contains all the orthopoxvirus ANK repeat genes detected in this genus in an intact form, sharing a >95% identity to their orthologues [34,40]. A comparison of the protein orthologues in other orthopoxviral species demonstrates that there is frequent loss or truncation of these genes, leading to a wide variation in the number of ANK repeat genes within each species, and their respective protein size, and possibly function [40]. Indeed, the inclusion of all fragmented ANK repeat proteins present in cowpox Brighton Red strain increased the number of possible orthologue groups to fifteen [34], with only one ANK repeat protein orthologue that lacks an F-box, represented by CPXV-041 and vaccinia virus K1L proteins (orthologue group X, [34]), being present in some form in all orthopoxvirus genomes examined [40]. A decrease in the number of ANK repeat proteins encoded within a poxviral genome is also matched by a decrease in the overall size of the genome. Variola virus has the smallest orthopoxvirus genome and contains only four intact ANK repeat proteins complete with their F-box domains [34,40]. This decrease in overall gene number, and intact ANK/F-box proteins also correlates with a decrease in host-range, from the broadest in cowpox virus to the narrowest in variola virus [92,93]. Loss of ANK repeat proteins and other immunomodulatory proteins is also seen in the highly attenuated and host-range limited modified vaccinia virus Ankara strain (MVA) [94], where only the highly conserved 68k ANK/F-box protein is retained [82]. The correlation of several ANK repeat proteins with host-range effects, and their loss in attenuated genomes suggests that these proteins play pivotal roles in host-range of poxviruses.

The ANK/F-box proteins within the orthopoxviruses and avipoxviruses, both intact and truncated, are spread extensively through both terminal regions of their genomes. Analysis of truncation events and horizontal transfer events in poxviruses demonstrates that these occur wholly within these terminal regions, this enables the fine tuning of the poxvirus relationship to particular host species without disruption of the conserved proteins encoded in the central genomic region [92]. The larger number of ANK repeat proteins present in these genomes indicates a potentially broad host-range among these genera. However the arrangement and frequency of ANK repeat genes shifts significantly with the *Parapoxvirus* genus and those in the various genera of the LSG [34,40].

These otherwise disparate groups of genera show a close similarity in the distribution and organisation of the ANK/F-box proteins, with the majority of known species containing one ankyrin gene in the left terminus of the genome, and no more than four in the right terminal region. Exceptions to this arrangement are the bovine papular stomatitis parapoxvirus, which contains three ANK repeat genes in the left terminal region, and the leporipoxviruses that contain inverted terminal repeats, with a copy of the left terminus ANK repeat protein (M-T5 in myxoma virus) also present in the right-terminal region [31,36,95]. Although the synteny of these genes is very similar across these genera the underlying DNA and amino acid sequence varies significantly between the *Parapoxvirus* and LSG genera, and their individual ANK repeat proteins, thus belong to their own genera-specific orthologue groups. The locations of these genes are therefore the result of independent duplication events and adaption to their specific hosts, which have either preserved an inherent synteny or arrived at one by convergence. However, despite the conservation of gene position and protein form, it is not known if their specific functions are conserved between species and genera.

Interestingly the right terminus of orthopoxvirus genomes also encodes a maximum of four ANK/F-box proteins, not counting duplications of orthologue groups I and II ANK repeat proteins located in the inverted terminal repeats. Group I duplicated proteins are present in most orthopoxviruses in both termini except variola viruses, whilst duplicated group II proteins are more restricted in intact forms in cowpox viruses and camelpox viruses or present as truncated proteins in several other species [34,40]. The left genomic terminus appears less restrictive and shows greater expansion of ANK repeat proteins, with eight proteins being present in cowpox viruses in addition to the two in the left inverted terminal repeat. The exception to this is the host-restricted variola virus that lacks an intact ANK repeat protein in the left genome terminus, and has four ANK repeat proteins present in the right genome terminus [27,40]. Extreme host restriction is only known in humans with one other poxvirus, molluscum contagiosum virus, which, like variola virus, has no other apparent host or animal reserve. As molluscum contagiosum virus also lacks all ANK repeat proteins including K1L it has presumably lost copies of the ancestral ANK repeat proteins that were preserved in the earlier divergence of the avipoxviruses [41,92,93]. This situation also appears to have occurred with crocodilepox virus and the squirrelpox virus. These three chordopoxvirus species that lack ANK repeat proteins also have a high GC% content, and have a limited known host-range; though for crocodilepox and squirrelpox virus this may be due to a paucity of information around their host-range rather than reflecting reality [42,43,96].

The origin of the poxviral ANK/F-box proteins is not known. Their distribution and phylogenetics indicate a shared ancestral gene prior to the splitting of the *Avipoxvirus* genus lineage, but likely after the division of the *Entomopoxvirinae* and *Chordopoxvirinae* sub-families. Many poxviral proteins, ankyrins included, have a higher similarity with eukaryotic rather than bacterial or viral proteins [97], with the ANK repeat proteins of cowpox virus strain GRI showing higher similarity to those from *Trichomonas vaginalis* compared to their closest viral scores from *Acanthamoeba polyphaga* mimivirus. The domain promiscuity usually associated with eukaryotic ANK repeat proteins, and also seen with in bacterial examples, has not been continued with the poxviral versions, with only the F-box motif being found as part of the ANK repeat protein [70,71]. Both of these features would thus appear to have been combined in ancestral proteins to enable their similar and widespread occurrence throughout the majority of the *Chordopoxvirinae* sub-family.

The poxviral F-box/PRANC domain was initially thought to be limited in distribution amongst the poxviral ANK repeat proteins [71], however, examples of PRANC domains with *N*-terminal ANK repeats have since been demonstrated in several intracellular bacterial species [70], and also in some of the host insect species these bacteria are associated with, such as the *Nasonia* and *Cotesia* jewel wasps [98]. As with poxviruses these bacterial and eukaryotic ANK/F-box proteins are encoded in multiple copies, often with additional paralogues present and with their master genes demonstrating a similarity in sequence indicating that they may also represent individual clades of orthologues [98,99,100]. Analysis of the *O. tsutsugamushi* F-box motifs show these have sequence similarities to poxviral F-boxes and are also truncated. The *Orientia* ANK/F-boxes can associate with the SCF1 ubiquitin ligase complexes, and also with the eukaryotic elongation factor 1α (EF1α) [100], and subsequently mediate its ubiquitination and degradation in several cell-lines, with one ANK/F-box protein co-localising EIFα with Cul1 to the nucleus. EIFα has a prominent role in protein translation but how this interaction and degradation would be of an advantage to the *Orientia* infection is yet to be explored. It is not known how the distribution of ANK/F-box proteins amongst poxviruses, intracellular bacteria and insects has occurred. The obligate relationship of rickettsial species, such as *Orientia* and *Wolbachia* with insects may be sufficient to enable horizontal transfer of ANK repeat proteins into their genomes such as seen with *Nasonia vitropenis* and *Cotesia congregata* [98]; however, there is no known association of chordopoxvirus species with these intracellular bacteria or their host wasps.

The presence of ANK/F-box proteins within poxviruses and also intracellular bacteria may indicate a convergent evolutionary adaptation to their shared environment. Alternatively this may reflect a shared host environment prior to the differentiation of the chordopoxviral genera. The strongest candidates for this latter scenario would be the entomopoxviruses, however these lack ANK repeat proteins and it is not known whether the entomopoxviruses contained ANK repeat genes and have since lost them, although this seems unlikely since ANK repeat genes are maintained in the rickettsial insect symbionts. The combination of ANK repeats with an F-box motif has been detected in a variety of organisms, particular eukaryotic and DNA viruses, although the vast majority of these are only as predictions (Uniprot/Pfam). However, many of these examples, such as those found in the giant DNA viruses of *Acanthamoeba*, are similar to eukaryotic F-box proteins having an *N*-terminal F-box with a *C*-terminal ANK repeat region [70], and are not identified with the poxviral and rickettsial F-box/PRANC domains. Horizontal transfer of genes from hosts is believed to account for many of the poxviral homologues to eukaryotic proteins that now function as immunomodulatory proteins [93,97]. Analysis of some of these genes, such as the poxviral interleukin-10 (vIL-10) suggest that similar viral genes are derived from separate integrative events in different viral/host relationships; for example, the vIL-10 genes of BPSV and orf virus show less similarity to each other and more to their respective host genes [13,93] even though both viral genes are located in the same relative position in their respective genomes, which is between the two ANK repeat proteins of orf virus (OV126 and OV128) and their BPSV orthologues. However the phylogenetic analyses [34,40,93] indicate that each of the poxviral ANK repeat proteins are derived from duplication events, rather than multiple horizontal transfers of host-specific ANK repeat proteins, and indeed no viral ANK repeat protein has been shown to have sufficient similarity with a host protein to be considered a homologue.

The advantage for maintenance of multiple yet subtly different ANK/F-box genes within poxviral genomes is not yet known, however several studies have illustrated the importance of the complete set of ankyrin proteins to a successful infection. This has been shown with myxoma virus where *in vivo* studies showed increased viral attenuation with knockout of ANK repeat protein M-T5 in comparison to knockouts of the ANK/F-box proteins M148R, M149R, and M150R, [84,95,101] reflecting a more potent role for M-T5 in enabling a virulent and systemic infection. A prominent role in virulence and host-range for the ANK repeat proteins is also shown by the characterisation of attenuated strains generated by extensive cell-passage, which frequently and apparently preferentially, result in deletion or mutation of ANK repeat proteins, as shown with attenuated strains of myxoma virus, sheeppox virus, fowlpox virus, and orf virus [33,101,102,103].

## 6. Role of Poxviral Ankyrin Proteins

The most significant amount of work with poxviral ANK repeat proteins have been in eliciting their interactions with other proteins and so illuminating the role they play in viral infection. This has shown that the ANK repeats often have effects on poxvirus host-range, while the F-box mediates interaction with the cellular SCF1 ubiquitin ligase. The influence of the role of ANK repeat proteins with poxviral host-range and permissive replication has been most studied using the vaccinia virus K1L ANK repeat protein that lacks an F-box. Initial research demonstrated that the absence of K1L led to host-range restriction in human cells [104] and not rabbit cells [89]. Further exploration of the difference in the K1L permissive effect discovered the C7L host-range factor, which together with K1L and the cowpox host-range protein, CP77, demonstrates a range of permissive behaviour on different cell-lines. The K1L, C7L and CP77 proteins demonstrate functional equivalence yet display a hierarchy in host-range in different cell-lines [90]. While CP77 is predicted to contain an ANK repeat region, complete with a functional F-box, the structural basis of C7L function remains unknown. As with K1L, these C7L and CP77 proteins are not present in all poxviral genera, possibly reflecting the wide variety of hosts infected. This variation is also shown with the K1L protein distribution amongst the orthopoxvirus genus, where the variola, camelpox, and taterapox viruses contain mutated versions. Although these three species are part of a monophyletic clade each of their K1L genes contains alternate premature stop-codons and multiple insertions and deletions, demonstrating independent mutation of this ANK repeat gene [40].

As K1L is composed entirely of ANK repeats its host-range functions are thus mediated by protein-protein interactions. The K1L host-range function was successfully mapped to residues within the ANK repeat 2 and 3 motifs that are situated on the protein’s convex surface, whilst the ANK repeat 4 and 5 motifs were required as structural, but not residue specific, features (Figure 2) [58,59]. However, so far only one protein, ACAP2, a GTPase activating protein, has been shown to bind to K1L in both rabbit and human cells. ACAP2 though, is not essential for successful replication in permissive cell-lines, but does require the ANK 5 repeat to bind to K1L, showing that different regions of the K1L protein may contribute to different aspects of its overall interaction with the host [105,59]. The influence of the K1L protein on the cellular anti-viral response was further demonstrated by its antagonism of interferon-β stimulated effectors in permissive human cell-lines, with this effect again mapping to the ANK 2 repeat and correlating with the mutations in host-range [106].

Additional work has also identified the influence of K1L on NF-κB function by inhibition of IκBα (inhibitor of κB) degradation [107], along with inhibition of auto-phosphorylation of the key anti-viral protein PKR (protein kinase RNA-dependent) and the subsequent lack of active PKR phosphorylation of eIFα2 (eukaryotic translation initiation factor 2), thus preventing eIFα2 mediated shut-down of protein synthesis [108,109]. The antagonism of K1L against the PKR pathway was further identified as a reduction in levels of virally produced, and previously unsuspected, early double-stranded mRNA. The function of the K1L associated decrease of this early mRNA was proposed as a facilitator for viral protein translation, thereby preventing the double-stranded mRNA activating PKR and stimulating anti-viral pathways prior to the viral E3 protein antagonism of PKR function; this effect also showed a host-range bias evident in rabbit RK13 cells preventing early transcript PKR activation, whilst only inhibition of intermediate transcript activation of PKR was observed in human HeLa cells [109].

The K1L ankyrin protein therefore interacts with cellular host-factors and helps contribute to the permissive state in certain species. The pivotal role of PKR activation in this response to viral infection means that many poxviruses also encode additional host-range proteins that antagonise this; the E3L protein family sequesters viral double-stranded RNA to prevent its activation of PKR, while K3L proteins bind activated PKR preventing its interaction with eIFα2. Again, these proteins are not evenly distributed amongst the different poxviral genera, varying from all three proteins present in the majority of orthopoxviruses, to only E3L being retained in parapoxviruses and squirrelpox virus, and all three being absent from molluscum contagiosum virus, crocodilepox virus, and avipoxvirus species [19,40,43]. It is not known why multiple antagonism of PKR is required in some species and not others, but this mirrors the deployment of numerous poxviral proteins against the NF-κB signalling and transcription system [110,111], indicating that some species may more extensive suppression of host response than others. The contribution of how these various host-range and virulence factors correlate to the different levels of tropism seen between different poxviruses is still being uncovered [112].

While investigations into K1L function have uncovered details of variable host-ranges, the focus on poxviral ANK/F-box proteins has been mainly on how they interact with the cellular ubiquitination pathway. The key interaction of the cellular F-box protein is with the Skp1 protein, and thus Skp1’s binding partner, Cul1, the supporting scaffold for the multi-protein E3 SCF1 ubiquitin ligase complexes (Figure 3) [113,114]. Initial indications of a connection between the poxviral ANK repeat proteins and the SCF1 ligase were shown by bioinformatic analysis that identified the *C*-termini of poxviral ANK repeat proteins as a truncated homologue of the cellular F-box [71], and by the demonstration of an interaction between the myxoma ANK repeat protein M-T5 and Cul1 [115]. The interaction between these poxviral F-box domains and the SCF Skp1 adaptor was then demonstrated with the ANK repeat proteins of the orf virus (strain NZ2) [78]; this interaction was also shown to extend to that of the larger Cul1 E3 ubiquitin ligase complex via the Skp1 adaptor protein. Binding by pull-down assays with Skp1 has since been demonstrated for myxoma virus proteins M-T5, M148R, M149R and M150R [84,85], ectromelia virus proteins EVM002, EVM005, EVM154 and EVM165 [83,116], cowpox virus CP77 (CPXV-025) [79], the 68k protein of vaccinia virus [82], and the G1R ANK repeat protein of variola virus, along with its homologues from cowpox virus (CPXV-006) and monkeypox virus (MPXV-003) [80]. Despite the prevalence of these proteins in the *Avipoxvirus* genus only one, FPV014, has a proven interaction with Skp1 (Table 3) [117].

**Table 3:** Poxviral ANK/F-box proteins and their described interaction partners. Known interacting proteins include key components of the SCF1 ubiquitin ligase complex (Skp1 and Cul1), with other known protein interactions also shown.

Genus	Species	Protein	SCF1 Interaction		Other Interaction	Host Range	Ref.
OPXV	ECTV	EVM002	Skp1		p105		[83,116]
		EVM005	Skp1	Cul1			[83,116]
		EVM154	Skp1				[83,116]
		EVM165	Skp1				[116]
	CPXV	CP77	Skp1	Cul1	p65	Yes	[79]
			Skp1		HMG20A		[118]
		CPXV-006	Skp1		p105		[81]
	VACV	68k	Skp1	Cul1		Yes	[82]
	VARV	G1R	Skp1		p105		[81]
	MPXV	MPXV-003	Skp1		p105		[81]
LPXV	MYXV	M-T5	Skp1	Cul1	Akt	Yes	[85,115]
		M148R ^a^	Skp1				[85,119]
		M149R	Skp1				[85,119]
		M150R ^b^	Skp1	Cul1			[84,85,95]
PPOV	ORFV	OV008	Skp1	Cul1			[78]
		OV123	Skp1	Cul1			[78]
		OV126 ^c^	Skp1	Cul1			[78,120]
		OV128	Skp1	Cul1			[78]
		OV129	Skp1	Cul1	CAND1		[78]
AVPV	FWPX	FPV014	Skp1				[117]

^a^ localises to nucleolus; ^b^ localises to nucleus with NF-κB; ^c^ localises to mitochondria. PPOV, *Parapoxvirus*; LPXV, *Leporipoxvirus*; OPXV, *Orthopoxvirus*; AVPV, *Avipoxvirus*. ORFV, orf virus; MYXV, myxoma virus; ECTV, ectromelia virus; CPXV, cowpox virus; VACV, vaccinia virus; VARV, variola virus; MPXV, monkeypox virus; FWPX, fowlpox virus.

The presence of a functional F-box-like motif and the experimental evidence of interactions with Skp1 and subsequently with Cul1, indicates that the poxviral ANK/F-box proteins target cellular SCF1 complexes. However, the link between their interactions with the SCF1 complex and the role of these proteins in viral infection and replication remains unclear. A number of poxviral ANK repeat proteins show functional effects that do not require the F-box, for example, the 68k ANK/F-box protein interacts with the SCF1 complex, but also has an essential F-box independent role in maintenance of viral DNA replication [121]. The cowpox CP77 ANK/F-box protein interacts with both the SCF1 complex, and the p65 unit of NF-κB, inhibiting NF-κB signalling, however its host-range function is independent of these two interactions and again does not require the F-box [79]. This suggests that the protein binding nature of the ANK repeat itself can have a significant role in creating interactions beneficial to viral infection.

**Figure 4:**
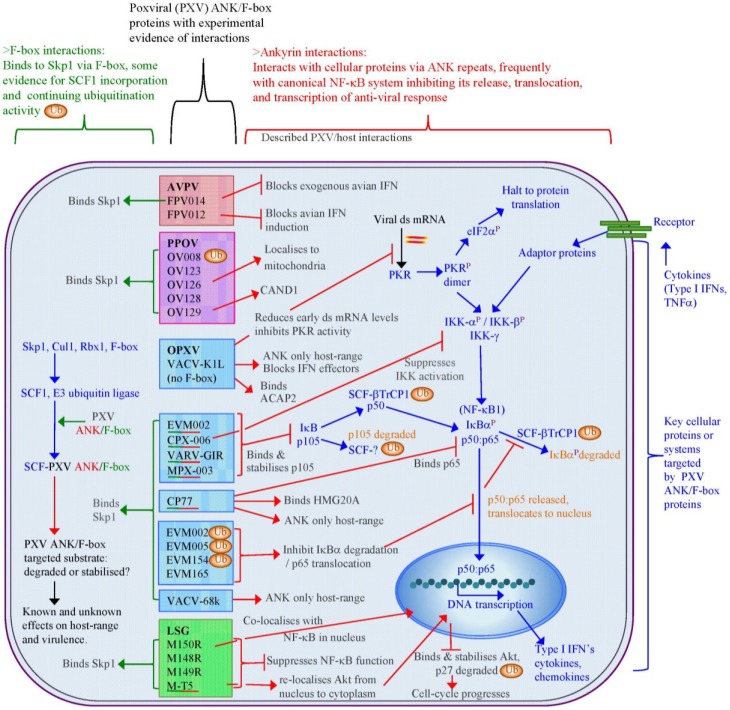
Interactions of ANK/F-box proteins are known from several poxviral species of the *Avipoxviru*s (AVPV) [117,122], *Parapoxvirus* (PPOV) [78], *Orthopoxvirus* (OPXV) genera [79,80,81,82,83,106,109,115,116], and also the *Leporipoxvirus* super-group (LSG) genera [84,101,123]. These poxviral proteins bind to Skp1 via F-box domains, and in some cases also interact with other cellular proteins or have an effect on other cellular systems, most frequently the canonical activation of NF-κB1 transcriptional regulation. Green/red underlined poxviral (PXV) proteins indicate demonstrated binding of both Skp1 and a cellular substrate. Ubiquitination (Ub) with SCF1 ligases containing a poxviral ANK/F-box has been shown in several examples: OV008, EVM002, EVM005, and EVM154; E3 ubiquitin ligases also control key parts of the NF-κB signalling via SCF-βTrCP1 and an unknown SCF that target p105 and IκBα for proteasomal degradation and processing.

There is also little direct evidence of poxviral ANK/F-box ubiquitination of target proteins, although active ubiquitinating SCF ligases, which contain poxviral ANK/F-box proteins, have been demonstrated (Figure 4). The orf virus OV008 protein produces an equivalent polyubiquitination signal to Skp2, a cellular F-box, when these proteins are expressed with SCF1 components, indicating that OV008 is unlikely to inhibit ubiquitination [78]. The interaction of poxviral ANK repeat proteins with a complete SCF1 ligase complex has been explored with ectromelia virus, myxoma virus, cowpox virus, and vaccinia virus [79,83,85,121] with *in vitro* ubiquitination also demonstrated with ectromelia proteins EVM002, EVM005, and EVM154 [83]. The network of the proven poxviral ANK/F-box proteins interactions detailed in Figure 4 shows that the interaction with the SCF1 complex via Skp1 and F-box binding is a highly likely scenario for this family of proteins. This may connect the degradative nature of the SCF1 complex with interference in other cellular functions, of which the NF-κB system is appearing as the leading candidate.

## 7. Poxviral ANK Repeat Protein Interaction with the NF-κB System

The NF-κB transcriptional pathway is a key target for viral suppression of the immune response [124,125]. The detection of viral infection initiates degradation of phosphorylated NF-κB inhibitors, IκB’s, by SCF ligases, releasing the sequestered NF-κB dimers, leading to the transcriptional activation of anti-viral gene expression (Figure 4) [126]. Many poxviral NF-κB directed proteins have already been functionally characterised [110,112] and shown to target multiple points within the NF-κB signal transduction pathway. This includes producing extracellular viral receptors to block signalling of interleukins (IL) and cytokines, such as IL-18, IL-1β, and tumour necrosis factor α (TNFα), intracellular blocking of PKR activation, inhibition of signal transduction within the cell, and prevention of the processing and release of sequestered NF-κB dimers, and their translocation to and activity within the nucleus [111,125]; unlike other viral species no poxviral activators of NF-κB have yet been discovered [124]. The poxviral anti-NF-κB proteins vary significantly between poxviral genera and species, and may be key virulence factors. For instance orf virus, and other parapoxviruses, contains a homologue of the vaccinia viral protein, E3L (orf virus protein OV020), which inhibits PKR binding to double-stranded mRNA, thus suppressing NF-κB activity by avoiding activation of the IκB kinase (IKK) complex [127], but these viruses lack any other homologues to known NF-κB antagonists from other poxvirus species. Instead orf virus expresses three proteins, unique to the *Parapoxvirus* genus, OV002, OV024 and OV121 that target different aspects of the NF-κB system, but are not equal in their effect on the host as only OV121 was shown to be a virulence factor while OV024 and OV002 have little effect on disease progression [128,129,130].

This multi-layered approach appears typical of poxviral control of NF-κB regulation and extends to the poxviral ANK repeat proteins, with the K1L protein inhibiting IκB degradation, [107] and thereby blocking NF-κB signal transduction of viral infection from Toll-like receptors, TLR2, TLR4 and TLR9 [131]. The role of the poxviral ANK/F-box proteins has also begun to show that these too have a direct influence on NF-κB activity as shown in Figure 4. However, the functional relationship between the ANK repeat and F-box domains with regards to NF-κB is not clear cut as no poxviral ANK/F-box protein has been directly demonstrated to be involved in ubiquitination of a NF-κB component or regulatory protein.

The G1R ANK/F-box protein of variola virus interacts with both Skp1 and NF-κB/p105 as demonstrated by yeast two-hybrid screening using VARV-G1R as the bait [80]. This interaction was also assessed with intact homologues of VARV-G1R including EVM002 (ectromelia virus) MPXV-003 (monkeypox virus) and CPXV-006 (cowpox virus) which all showed the same effect by binding the p105 precursor of the NF-κB p50 sub-unit, and preventing formation of active NF-κB. Surprisingly the combination of the SCF1 and p105 substrate did not lead to p105 degradation but rather its stabilisation, which may indicate separate roles for these two interactions rather than a re-direction of proteasomal degradation by the poxviral ANK repeat proteins [80].

Knockout studies on the functional role of the cowpox ANK repeat protein CPXV-006, indicated by the association with p105, demonstrated a clear influence over regulation of NF-κB activity, where a CPXV-006 mutant was able to induce degradation of the NF-κB p105 sub-unit [81]. In addition, the CPXV-006 knockout mutant could successfully phosphorylate the IKK complex, activating this to enable the phosphorylation of IκBα, with its subsequent degradation, and the release and nuclear translocation of NF-κB, along with the transcription of anti-viral interferons (IFN) such as Il-6, IL-1β, and TNFα. Although there was no effect *in vitro* on viral replication in the cell-lines tested, the knockout’s effect on NF-κB was reflected in increased inflammation response in mouse animal models demonstrating that this protein could successfully repress stimulation of NF-κB induced immune response [81].

Further details on the role of ANK/F-box protein function in the NF-κB system have been indicated by knockout and individual expression experiments of ANK repeat proteins from ectromelia and myxoma viruses [101,116]. Ectopic expression of ectromelia ANK/F-box genes confirmed that their proteins each associate with Skp1, prevent TNFα stimulated degradation of IκBα and thereby inhibiting p65 nuclear translocation, and also that their F-box regions are required for this effect. However, knockout of each of these genes from ectromelia virus individually did not demonstrate a dominant inhibitory role for any with IκBα degradation and subsequent p65 translocation [116]. A double knockout of EVM002 and EVM005 still demonstrated inhibition of TNFα induced degradation of IκBα, indicating additional poxviral modulation of the NF-κB system was present beyond the function of these two proteins [132]. The EVM005 protein also demonstrated a virulence role in two mouse models, and intriguingly showed similar levels of virulence with or without the F-box, despite its requirement for inhibitory effects on NF-κB signalling *in vitro*, suggesting that the ANK repeat domain could effectively sequester its substrate and still contribute to virulence without a presumed EVM005 linked SCF1 ubiquitin ligase activity, in a similar manner to the F-box independent function of CP77 and 68k proteins [116,132].

In myxoma virus a double knockout of the two M-T5 copies showed that the M-T5 ANK/F-box protein has a greater impact on apoptosis and cytotoxicity *in vitro* in comparison to the three ANK/F-box proteins expressed from the right side of the genome (M148R, M149R and M150R) [95,101,119,123]. Deletion of M-T5 alone led to significant attenuation of disease with a small primary lesion and no dissemination of disease, however, all four myxoma proteins inhibited p65 accumulation in the nucleus [95,101]. These studies demonstrate the combined role that the ANK/F-box proteins have within the ectromelia and myxoma viruses, with the indication that each makes a contribution towards maintaining viral infection through interactions with SCF1 ligase complexes and inhibition of NF-κB signalling. However the larger contribution of M-T5 to disease progression and its known interaction with an additional binding partner, the serine/threonine kinase, Akt, indicates that the ANK/F-box proteins may also have individually specific roles in infection.

## 8. Other Poxviral ANK/F-Box Interactions

Information about ANK/F-box interactions beyond their effects on the NF-κB system is unfortunately more limited. The M-T5 myxoma virus protein is one of the few ANK/F-box proteins that have been shown to simultaneously bind Skp1 and a potential cellular substrate protein. M-T5 acts as scaffold linking Skp1 to the protein kinase, Akt, a key regulator of several pathways involved in apoptosis and cell growth [85,115,133]. Surprisingly though, this apparent connection to the SCF1 ubiquitin ligase via Skp1 leads to a stabilisation of Akt levels, rather than their decrease due to M-T5/SCF1 mediated degradation; the M-T5 bound Akt can therefore continue to phosphorylate cellular targets such as p27. Stabilised Akt and phosphorylated p27 enable continued cell-cycle progression through the G0/G1 check-point, presumably avoiding programmed cell-death as a response to viral infection, and also enabling a switch to a permissive state for myxoma infection for some previously non-permissive human cancer cell-lines [115,133]. Akt was also shown to be essential for successful myxoma virus infection and late gene expression in one of the M-T5-dependent permissive cancer cell-lines [133]. M-T5 was further shown to be a functional homologue to the cellular PIKE-A protein (P13 kinase enhancer activating Akt), which has low sequence identity to M-T5 but does contains several ANK repeats [134]. M-T5 ANK repeats 1 and 2 were shown to be necessary for the binding to Akt and the bridging of this with Skp1. Interestingly, Akt was not detected in an M-T5 yeast two-hybrid screen of myxoma infected cells, although Skp1 was along with 12 other cellular proteins, whose interaction has yet to be confirmed [85].

Yeast two-hybrid studies also identified a binding partner for the cowpox virus ANK/F-box CP77 protein with the chromosome remodelling protein, HMG20A [118]. CP77 associates with HMG20A in the viral factory of infected CHO-K1 cells and is necessary for HMG20A dissociation 8 h post-infection. However, the CP77 PRANC domain was not required for this association, which could be further localised to CP77 ANK repeat 5, the removal of which prevented HMG20A dissociation from the viral factory and correlates with poor viral replication [118].

There are as yet no reports of parapoxvirus ANK/F-box interaction partners or affected pathways. However, the OV126 ANK/F-box protein has been demonstrated to localise to mitochondria via two ankyrin repeats (ANK 8 and ANK 9); these ANK repeats were essential for this localisation, but no detectable apoptotic effect was observed [120]. Interaction with the SCF ligase regulatory protein, CAND1, was also demonstrated using a cellular pull-down with OV129 ectopic expression in mammalian cells [78]. Although CAND1 was initially believed to be responsible for inhibition of SCF activity by sterically blocking Skp1 binding, recent studies have demonstrated that it is involved in the exchange of different substrate receptor proteins [135]. The OV129 pull-down may therefore have captured a state where this exchange of substrate receptors was ongoing. Interestingly this also raises the question of the poxviral F-box substrate receptor affinity for Skp1, and how these compete with cellular F-boxes and the multiple F-box proteins expressed by the poxvirus.

Limited information is available on the role of the ANK/F-box proteins in avipoxviruses, with only two of the many versions present in fowlpox virus having been investigated during the course of a study of the effects of avian interferon on fowlpox virus infection. This determined that two ANK repeat proteins made a contribution to the high resistance of fowlpox virus to avian interferon: FPV012, blocks induction of chicken IFN-2 (ChIFN2), and FPV014 acts by enhancing the resistance to exogenous ChIFN-2. Despite both ANK repeat proteins having predicted PRANC domains, interaction with Skp1 and Cul1 could only be shown for FPV014 [117,122]. A canarypox virus protein, CNPV030, identified as an orthologue of FPV012 [34], could also successfully inhibit ChIFN-2 induction [122], indicating that avipoxvirus ANK repeat proteins may also show conserved functions across their orthologue groups.

Little is known about the regulation of different poxviral ANK/F-box proteins. In orf virus early transcription has been shown for OV008 in NZ2 strain [136], and also detected for OV128 and OV129 in a second strain Orf-11 [137] whilst for the other orf ANK/F-box proteins, the timing of transcription remain unknown. Varied transcription times are known from other poxviruses, myxoma virus M-T5 and M150R are early, while M148R is late, and M149R is intermediate [115,119,123]. Ectromelia virus ANK/F-box proteins are mainly expressed early with only EVM154 distinguished as a late protein [116]. Early transcription also observed for the two fowlpox proteins FVP012 and FVP014 [117,122] Localisation of the ANK/F-box protein can also be varied. While most interactions with Skp1 and SCF1 are detected in the cytoplasm, several ANK/F-box proteins can localise to the nucleus, in particular M150R, and to some degree M-T5 [115,123,133].

The interaction of ANK/F-box proteins with ubiquitin ligases and their potential targets is therefore slowly being revealed with the most detail developing within the context of NF-κB pathway modulation. However, the poxviral repertoire of proteins that affect or act as ubiquitin ligases extends beyond the ANK/F-box proteins covered here with many poxviruses possessing a variety of alternative proteins that may exploit ubiquitin ligase activity in different ways. For instance, the *Orthopoxvirus* genus and LSG group contain poxviral BTB substrate adaptors that can interact with the Cul3 containing ubiquitin ligases and affect virulence and host-range [138]. Functional information on these interactions is more limited than ANK/F-box proteins but the ectromelia EVM150 BTB protein has been clearly shown to inhibit NF-κB translocation but independently of both Cul3 and its own substrate binding Kelch domain [139]. The poxviral specific p28 ligases are known virulence factors found in the majority of poxviral genera, and contain a DNA binding domain and a RING domain [19,138]. The p28 ligases co-localise to the viral factory with ubiquitin and are regulated by proteasomal degradation [140]. Poxviral versions of MARCH (Membrane associated RING-CH) ligases are also present in the LSG group, where a key example is the myxoma viral M153R protein that enables the internalization and lysosomal degradation of CD4 and removal of cell surface MHC class I [138,141]. The *Parapoxvirus* genus along with the crocodilepox virus, molluscum contagiosum virus, and squirrel poxvirus species all lack these additional ubiquitin ligases. However, these viruses do contain a RING based protein that lacks ubiquitin ligase activity and has sequence similarities to APC11, a component of the anaphase-promoting complex (APC). In orf viral infections this protein, poxvirus anaphase promoting complex/cyclosome regulator (PACR) competes with APC11 and impairs APC’s ubiquitin ligase activity [142,143].

In addition to these described interactions the continued function of cellular ubiquitin ligases and proteasomes in poxvirus-infected cells are being identified as fundamental aspects of poxviral replication. The presence of a functioning proteasome has also been shown to be required for successful vaccinia viral DNA replication and late gene expression, with an essential role in uncoating of the virion particle [144,145,146]. The vaccinia viral core proteins were shown to be K48 poly-ubiquitinated during assembly of the virion, enabling their proteasomally mediated degradation during uncoating of the virion genome in a subsequent infection [146]. Although this suggests that ubiquitin ligase activity is required for production of a viable virion this has not yet been directly confirmed or a specific ligase identified. However, siRNA screening showed that Cul3 based ubiquitin ligases, and not the Cul1 SCF complex or ANK/F-box proteins were involved with enabling poxviral DNA replication [146]. An active proteasome is also required for the dissociation of the vaccinia virion core lateral bodies and the activation of the essential poxviral VH1 dual-purpose phosphatase that packs within the lateral bodies; VH1 dephosphorylates several viral proteins and also STAT1 thereby mediating an initial inhibition of the interferon induced immune response [147].

These details indicate that viral mediated ubiquitination may not be simply directed to control of host protein stability, but also to ensure specific regulation of viral protein activity and regulation. The poxviral ANK/F-box proteins are the most widely distributed poxviral proteins that may influence cellular ubiquitin ligase activity and therefore could have roles outside of the known connections to down-regulation of NF-kB signaling, supporting other essential aspects of their host-range and life cycle.

## 9. Conclusions

The poxviral ANK/F-box proteins demonstrate a number of conserved features that indicate a shared evolutionary background and role. Poxviral genomes that encode ANK repeat proteins demonstrate a maintenance of a minimal number of ankyrin proteins localised to the terminal regions of the genome, a frequently conserved synteny of these genes between genera, and a segregation into distinct orthologue groups due to gene duplication within and between genera. With the exception of one of these orthologue groups, that contain the ANK repeat K1L protein, all intact proteins have a consistent arrangement of an *N*-terminal ANK repeat domain and a *C*-terminal F-box, which have sequence and functional similarity to cellular F-box domains. The majority of poxviruses thus have the potential to interact with the SCF1 ubiquitin ligase complex through a variety of substrate receptors.

Regulation of the SCF1 complex is finely controlled by a variety of proteins and additional complexes that act to block substrate binding, deubiquitinate the complex, and exchange the substrate receptors to re-target the complex. The poxviral ANK repeat proteins must therefore compete within this dynamic and tightly regulated interplay to ensure their effective engagement with the SCF1 complex. While the outcome of this might be expected to be a re-focusing of the infected cell’s degradative capability to the poxviral targets, examples so far have indicated that targeted host proteins, which can act as control points in the anti-viral response (e.g., Akt in the cell-cycle and p105 in NF-κB regulation), are stabilised and protected from the degradation by cellular activity induced by viral infection, thereby maintaining the infected cell in a state conducive to viral replication. The maintenance of a variety of ANK/F-box proteins, often with a redundancy in their effects, within most poxviral species demonstrates the importance of these proteins in viral infection.
